# Complicated Acute Pericarditis and Peripheral Venous Catheter-Related Bloodstream Infection Caused by Methicillin-Resistant *Staphylococcus aureus* after Influenza B Virus Infection: A Case Report

**DOI:** 10.1155/2023/4374552

**Published:** 2023-05-02

**Authors:** Fumihiro Ochi, Hisamichi Tauchi, Hiromitsu Miura, Tomozo Moritani, Toshiyuki Chisaka, Takashi Higaki, Mariko Eguchi

**Affiliations:** ^1^Department of Pediatrics, Ehime Prefectural Niihama Hospital, Niihama, Ehime, Japan; ^2^Department of Pediatrics, Ehime University Graduate School of Medicine, Toon, Ehime, Japan

## Abstract

**Background:**

In this study, we report the case of a 14-month-old female patient transferred from another hospital to our hospital with a 9-day history of fever and worsening dyspnea. *Case Report*. The patient tested positive for influenza type B virus 7 days before being transferred to our hospital but was never treated. The physical examination performed at presentation revealed redness and swelling of the skin at the site of the peripheral venous catheter insertion performed at the previous hospital. Her electrocardiogram revealed ST segment elevations in leads II, III, aVF, and V2–V6. An emergent transthoracic echocardiogram revealed pericardial effusion. As ventricular dysfunction due to pericardial effusion was not present, pericardiocentesis was not performed. Furthermore, blood culture revealed methicillin-resistant *Staphylococcus aureus* (MRSA). Thus, a diagnosis of acute pericarditis complicated with sepsis and peripheral venous catheter-related bloodstream infection (PVC-BSI) due to MRSA was made. Frequent bedside ultrasound examinations were performed to evaluate the outcomes of the treatment. After administering vancomycin, aspirin, and colchicine, the patient's general condition stabilized.

**Conclusions:**

In children, it is crucial to identify the causative organism and provide appropriate targeted therapy to prevent worsening of the condition and mortality due to acute pericarditis. Moreover, it is important to carefully monitor the clinical course for the progression of acute pericarditis to cardiac tamponade and evaluate the treatment outcomes.

## 1. Introduction

Acute pericarditis is an inflammation of the pericardium with or without pericardial effusion. This condition is rare in children. However, it can progress to pericardial effusion or even cardiac tamponade [1, 2]. In addition, in many patients with pericarditis, the etiology of the pericardial effusion cannot be determined. Such idiopathic pericardial effusions are presumed to be viral or postviral in etiology [1, 3–5]. The most common identifiable causes of acute pericarditis in children are bacterial infections, and the majority of such cases are attributed to bacteremia [4].

The management of acute pericarditis in children is determined by the severity of symptoms and the underlying cause. Although most patients are treated for a presumptive viral cause with nonsteroidal anti-inflammatory drugs and colchicine, antibiotic therapy and pericardial drainage are essential for managing children with bacterial pericarditis and purulent effusions [4, 6, 7].

Herein, we present the case of a 14-month-old girl having acute pericarditis complicated with PVC-BSI due to MRSA. She presented with a recent history of influenza type B viral infection and was successfully treated without pericardial drainage.

## 2. Case Presentation

A previously healthy 14-month-old female patient presented to nearby clinic with a history of high-grade fever lasting 2 days and cough in winter 2018 on day 2 of disease onset. Although she was diagnosed with influenza B infection using an influenza virus rapid antigen-detection test, no specific therapy was initiated in the clinic. Moreover, she had not received the influenza vaccine that season. Furthermore, on day 7 of onset, she was admitted to another hospital for persistent fever and cough, where a peripheral venous catheter was immediately inserted. Her chest X-ray revealed no abnormal findings, such as an increase in the cardiothoracic ratio. She was treated with intravenous rehydration and administered prednisolone (1 mg/kg/day) as an anticytokine therapy via the peripheral vein. However, her fever, cough, and dyspnea continued to worsen. Therefore, 2 days later, i.e., on day 9 of onset, she was transferred to our hospital.

On physical examination, she was found to be conscious but drowsy (Modified Glasgow Coma Scale for infants and children: E2V3M4). Her vital signs were as follows: body temperature, 39.1°C; blood pressure, 92/65 mmHg; heart rate, 158 beats/min; respiratory rate, 65 breaths/min; and oxygen saturation, 99% with O_2_ at 5 L/min via a mask. She had moderate difficulty in breathing, with suprasternal and subcostal retractions. Her capillary refill time was 3 s.

In our patient, although a pericardial rub was not present, muffled heart sounds were heard. Moreover, her respiratory sounds were diminished on the left lower side, with wet rales. There was no jugular vein distension, pedal edema, or pulsus paradoxus. Furthermore, phlebitis, including redness, swelling, and slightly purulent discharge oozing from the skin at the site of insertion of the peripheral venous catheter into the cephalic vein performed at the previous hospital, was noted.

Laboratory examination of the patient revealed the following: white blood cell count, 34200 cells/*μ*L (normal reference values: 4500–13500 cells/*μ*L), with 85.0% neutrophils; red cell count, 3.61 × 10^6^ cells/*μ*L (normal reference values: 3.76 × 10^6^–5.00 × 10^6^ cells/*μ*L); hemoglobin, 8.9 g/dL (normal reference values: 11.3–15.2 g/dL); hematocrit, 27.0% (normal reference values: 33.4%–44.9%); and platelet count, 35.9 × 10^4^ cells/*μ*L (normal reference values: 13.1 × 10^4^–36.9 × 10^4^ cells/*μ*L).

Inflammatory biomarkers were elevated: C-reactive protein, 29.75 mg/dL (normal reference values: <0.20 mg/dL); procalcitonin, 1.100 ng/mL (normal reference values: <0.5 ng/mL); lactate dehydrogenase, 453 U/L (normal reference values: 120–240 U/L); creatine kinase, 15 U/L (normal reference values: 45–226 U/L); B-type natriuretic peptide, 235 pg/mL (normal reference values: ≤18.4 pg/mL); and troponin-I, <10 pg/mL (normal reference values: ≤26.2 pg/mL).

**Figure 1:**
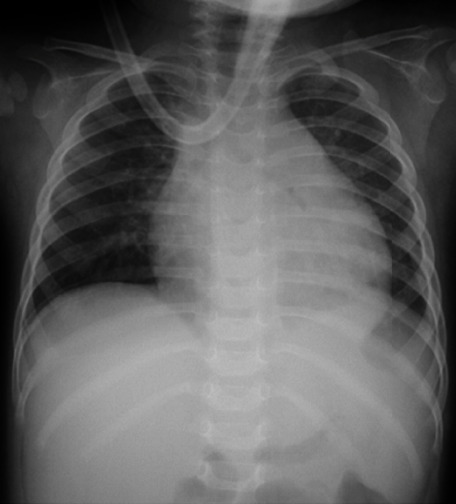
Chest X-ray obtained at admission showing an enlarged cardiac silhouette with a cardiothoracic ratio of 0.60.

**Figure 2:**
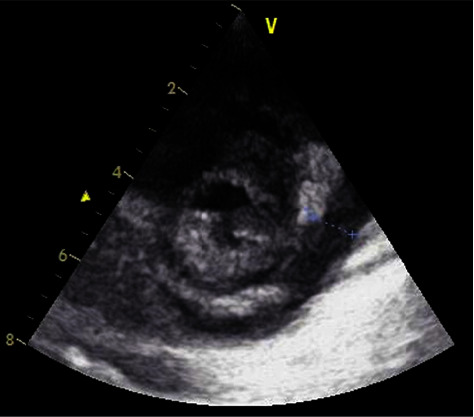
Parasternal short-axis view of transthoracic echocardiograms during the acute illness revealing 8.6-mm pericardial effusion thickness.

The chest X-ray revealed cardiomegaly, with a cardiothoracic ratio of 0.60 (Figure 1). A transthoracic echocardiogram revealed pericardial effusion, with no respiratory variations, biventricular dysfunction, or global hypokinesia (Figure 2).

**Figure 3:**
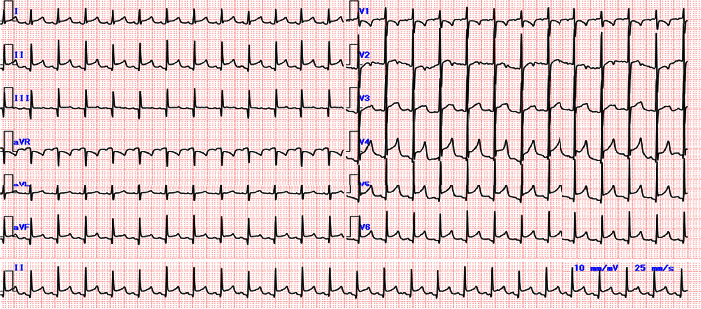
Electrocardiography findings on admission indicating sinus tachycardia, PR depression, and concave ST elevation in leads II, III, aVF, and V2–V6 as well as PR elevation and ST depression in aVR and V1.

**Figure 4:**
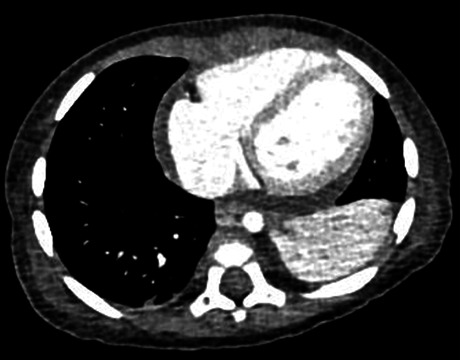
Contrast-enhanced computed tomography scan of the chest showing a pericardial effusion and left partial atelectasis.

The electrocardiography scan revealed PR depression and concave ST elevation in leads II, III, aVF, and V2–V6 as well as PR elevation and ST depression in aVR and V1 (Figure 3). Chest computed tomography scan revealed pericardial effusion and atelectasis with pleural effusion in the left lung (Figure 4).

In the present case, a diagnosis of sepsis, acute pericarditis, and PVC-BSI was made. For treating acute pericarditis, colchicine (0.02 mg/kg/day), aspirin (30 mg/kg/day), and diuretics were administered. After obtaining two consecutive blood culture samples from the peripheral vein and removing the peripheral venous catheter, cefotaxime (150 mg/kg/day) and vancomycin (VCM, 45 mg/kg/day) empiric therapy was started on day 9 of onset. Blood culture indicated only gram-positive cocci from the aerobic bottle 1 day after the culture. Mass spectrometry and microbial analysis revealed methicillin-resistant *Staphylococcus aureus* (MRSA) in the blood culture samples. The minimum inhibitory concentration for the isolate was >8 *µ*g/mL for ampicillin, 8 *µ*g/mL for sulbactam/ampicillin, 16 *µ*g/mL for cefazolin, 32 *µ*g/mL for ceftriaxone, ≤0.5 *μ*g/mL for gentamicin, ≤1 *μ*g/mL for VCM, and 0.5 *μ*g/mL for daptomycin. The same organism was found to grow from the tip of the peripheral venous catheter and skin swab culture at the site of insertion of the peripheral venous catheter. In the peripheral venous catheter tip, the colony count was >15 colony-forming units. We performed VCM de-escalation.

Additionally, frequent bedside transthoracic echocardiography examinations were performed to carefully monitor the patient for pericardial fluid and cardiac function. Her fever and dyspnea improved over the next day, and the size of the pericardial effusion decreased. The dose of the anti-inflammatory agent was tapered after the symptoms subsided.

In the following days, the pericardial effusion improved to that at baseline. Furthermore, the patient was discharged 2 weeks after starting intravenous antimicrobial therapy. The administration of aspirin (for 99 days) and colchicine (for 188 days) was discontinued in an ambulatory setting. During the 4-year follow-up, the patient developed no complication or recurrence.

## 3. Discussion

Here, we report the case of a 14-month-old girl who presented with acute pericarditis complicated with sepsis and PVC-BSI due to MRSA and a recent history of influenza type B viral infection. The influenza B virus was detected using the rapid antigen test, and MRSA was detected using the blood culture. Notably, PVC-BSI caused by MRSA rapidly deteriorated the general condition of the patient. Although both influenza B virus and MRSA are considered as rare causative organisms of acute pericarditis in children, pericarditis caused by these organisms leads to sepsis and is associated with significant mortality [1, 8]. In our patient, the pericardial effusion did not progress to cardiac tamponade, and the general condition became stable; hence, pericardiocentesis was not performed. Therefore, it is unclear whether the direct cause of the pericardial effusion was the influenza B virus and/or MRSA.

Based on the clinical history of the patient, including her response to treatment, we hypothesized that she first developed influenza B pericarditis and then developed secondary infection due to MRSA from PVC. It has been reported that many cases of acute pericarditis caused by the influenza virus improve after anti-inflammatory therapy, and the prognosis of such cases is good [9]. However, some studies have reported the cases of severe pericarditis with cardiac tamponade due to the influenza A virus [10, 11]. Although acute pericarditis caused by influenza B virus is rare, there have been few recent reports of severe acute pericarditis with large pericardial effusion and cardiac tamponade [12, 13].

Moreover, many cases of acute pericarditis caused by *S. aureus*, including MRSA, are complicated by bacteremia, leading to sepsis and cardiac tamponade. Such patients require surgical treatment [1]. Acute pericarditis due to *S. aureus* should be suspected in children with severe clinical illness or sepsis along with pericardial involvement and coexisting skin/soft tissue or musculoskeletal infection [1, 2]. Given that our patient also developed PVC-BSI due to MRSA, the possibility of acute pericarditis due to MRSA was always considered.

Ganji et al. reported the case of a patient with MRSA pericarditis who presented with cardiac tamponade and a recent history of influenza type A viral infection [14]. Moreover, there have been reports of bacterial pericarditis after viral infection.

Superinfection of both bacterial and viral infections can alter the actual clinical course of the disease. In particular, secondary bacterial infections following influenza infection substantially complicate the clinical course of the disease and increase morbidity and mortality in such patients [15]. Notably, susceptibility to secondary bacterial superinfections is typically observed approximately 1 week after influenza infection [16]. In the present case, the patient developed sepsis and PVC-BSI due to MRSA 9 days after the onset of infection caused by influenza B virus. These findings indicate the importance of determining the immune status, especially approximately 1 week after influenza infection.

Therefore, it is important to proactively identify the causative organism of infection and to carefully monitor the clinical course and host immune status.

Recently, many studies have attempted to elucidate the immunological mechanism of influenza infection, which is complicated by bacterial superinfection. Robinson et al. [17] reported that influenza virus inhibits bacteria-induced IL-1*β* production in the host and impairs the host defense against bacterial infections. Additionally, it is known that regulatory T cells produce IL-10 during influenza infection [18]. Furthermore, the produced IL-10 deactivates macrophages and decreases the production of cytokines involved in the host defense against bacterial infection by T cells [19]. Given that the production of the anti-inflammatory cytokine IL-10 increases during influenza virus-related bacterial superinfection, regulatory T cells may induce susceptibility to secondary bacterial infections [20].

In the case of acute pericarditis of unknown cause, it is desirable to prioritize the elucidation of the cause when the patient's general condition is stable as well as to perform frequent cardiac ultrasound examinations or bacterial cultures, e.g., blood cultures, and follow-up the patient carefully. If the patient's general condition is unstable, suppurative pericarditis should be considered first. In such patients, we recommend starting antibiotics immediately and performing surgical treatment, such as pericardiocentesis or diagnostic tap.

Gabler reported that bedside ultrasound examination might be a critical modality for more rapid assessment of pericarditis and cardiac tamponade secondary to infection. Moreover, bedside ultrasound examination in such a case could help prevent deaths and/or evaluate treatment modalities [21]. In our patient, it was difficult to determine whether the direct cause of the acute pericarditis was influenza B virus or MRSA; therefore, an assessment was made based on the clinical course of the patient. Our patient had PVC-BSI, and her clinical course was carefully monitored with frequent ultrasound examinations, assuming that acute pericarditis was caused by *S. aureus*. Furthermore, antibiotic therapy, peripheral venous catheter removal, and anti-inflammatory therapy with aspirin and colchicine led to a significant improvement in our patient's symptoms. Therefore, no surgical treatment was performed. Notably, the bedside ultrasound findings were very helpful in determining the course of treatment.

The duration of treatment depends on the presence of complications due to MRSA infection. Therefore, in patients with an unknown cause (virus or MRSA), the presence of osteomyelitis, infective endocarditis, or abscess should be checked. In our patient, we performed echocardiography, computed tomography, and several blood cultures; furthermore, taking into account the results of these tests in conjunction with clinical findings, the duration of antibiotic treatment was set at the duration of treatment for MRSA bacteremia.

Briefly, in children, it is crucial to identify the causative organisms and provide appropriate targeted therapy to prevent worsening of condition and mortality due to acute pericarditis. In addition, it is important to carefully monitor the clinical course for the progression of acute pericarditis to cardiac tamponade and evaluate the treatment outcomes.
